# Acute respiratory distress syndrome after spontaneous rupture of a large pulmonary hydatid cyst in a 17‐year‐old male: A case report

**DOI:** 10.1002/ccr3.7194

**Published:** 2023-04-12

**Authors:** Mehdi Salimi, Shirin Assar, Dena Mohamadzadeh, Asal Kanjorpor

**Affiliations:** ^1^ Clinical Research Development Center, Imam Reza Hospital Kermanshah University of Medical Sciences Kermanshah Iran

**Keywords:** ARDS, cyst rupture, pulmonary hydatid cyst

## Abstract

Pulmonary hydatid cysts (PHC) and their complications are still a health concern in endemic countries. Here we described a 17‐year‐old male presented with a large PHC with a spontaneous rupture. He developed acute respiratory distress syndrome (ARDS) requiring mechanical ventilation. He was treated with albendazole, broad‐spectrum antibiotics, and corticosteroids. The patient's general condition did not allow any attempt for surgical resection of the cyst. He was discharged in stable condition after one month and referred to a thoracic surgeon for resection of the cyst. As far as we know ARDS after hydatid cyst rupture was rarely reported, and through this case report we aimed to raise awareness of this possible life‐threatening complication.

## 1 INTRODUCTION

Acute respiratory distress syndrome (ARDS) is defined as a process of nonhydrostatic pulmonary edema and hypoxemia. Different etiologies and risk factors have been attributed to the development of ARDS including bacterial or viral pneumonia, sepsis, aspiration of gastrointestinal lumen contents, trauma, and blood transfusion. Bilateral chest opacities on chest X‐ray or CT scan are characteristic of ARDS. Supportive therapy with mechanical ventilation and fluid management is the cornerstone of treatment. Corticosteroids have been known as an effective therapy.^1^, ^2^ Pulmonary hydatid cysts caused by a parasitic tapeworm named echinococcus granulosus may cause cough, chest pain, hemoptysis, pneumothorax, pleural effusion, or empyema. Fever and hypersensitivity reactions including anaphylaxis may occur as a consequence of pulmonary hydatid cyst rupture.^3^

In this case report, we described a young male who developed acute respiratory distress syndrome after the spontaneous rupture of a pulmonary hydatid cyst. Hydatid cyst‐related ARDS is a rare and life‐threatening complication that is not still well recognized.

## 2 CASE PRESENTATION

A 17‐year‐old male presented to the emergency department of Imam Reza hospital of Kermanshah province of Iran. He complained of fever, repetitive cough, dyspnea, and chest pain initiated a few hours prior to hospital admission. He did not report any respiratory symptoms before this. His past medical history or drug history was unremarkable. He denied cigarette or alcohol abuse, and previous exposure to pets. He worked on a vegetable farm with his family. His familial history was unremarkable.

On general examination he was febrile (T = 38.5 degrees Celsius), His pulse rate was 120 beats/minute, his respiratory rate was 24, and his blood pressure was 110/70 mmHg. His oxygen saturation was 91% on room air. He was not in respiratory distress and did not use respiratory accessory muscles. On respiratory system examination, respiratory sounds were decreased on the right hemithorax. The rest of the clinical examination including the cardiovascular system and abdomen were within normal limits.

The results of laboratory tests on admission day were as follows: hemoglobin of 15.6 mg/dL, white blood cell count (WBC) 12.7 × 10^3^/mm^3^ (differential count: neutrophils 90%, lymphocytes 7%, and monocytes 3%), platelet count 229 × 10^3^/mm^3^, creatinine 0.9 mg/dL, International normalized ratio (INR) 1, partial thromboplastin time (PTT) 28 s, lactate dehydrogenase (LDH) 622 IU/mL (normal:225–500 IU/mL), aspartate transaminase (AST) 62 IU/L (normal:5–40 IU/L), alanine transaminase (ALT) 97 IU/L (normal:5–40 IU/L), alkaline phosphatase (ALP) 123 U/L (normal:80–306 U/L), total bilirubin 0.7 mg/dL (normal:0.2–1.4 mg/dL), direct bilirubin 0.3 mg/dL (normal:0–0.4 mg/dL), erythrocyte sedimentation rate (ESR) 10 mm/hr, and C‐reactive protein (CRP) was positive.

**FIGURE 1:**
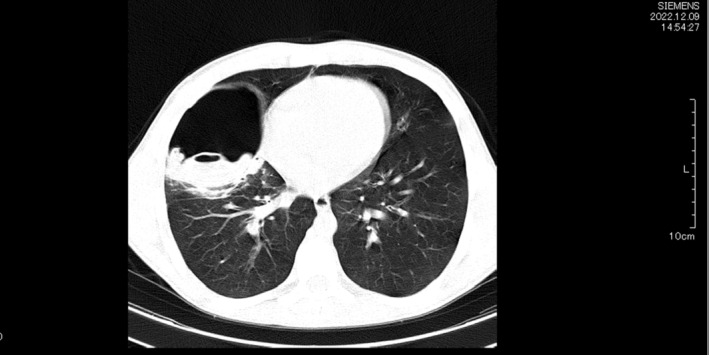
Chest CT scan showing a large cavitary lesion on the middle lobe of the right lung which contains a large amount of air, and soft tissue resembling a membrane (Water Lily sign).

A chest computed tomography (CT) scan was performed and showed a large cavitary lesion on the middle lobe of the right lung which contained a large amount of air, and soft tissue resembling a membrane. The imaging findings were compatible with the hydatid cyst. Few ground glass opacities were observed in the lower lobe of the right lung suggesting rupture of hydatid cyst. The left lung had no pathologic lesion (Figure 1). Ultrasonography of the abdomen was normal.

**FIGURE 2:**
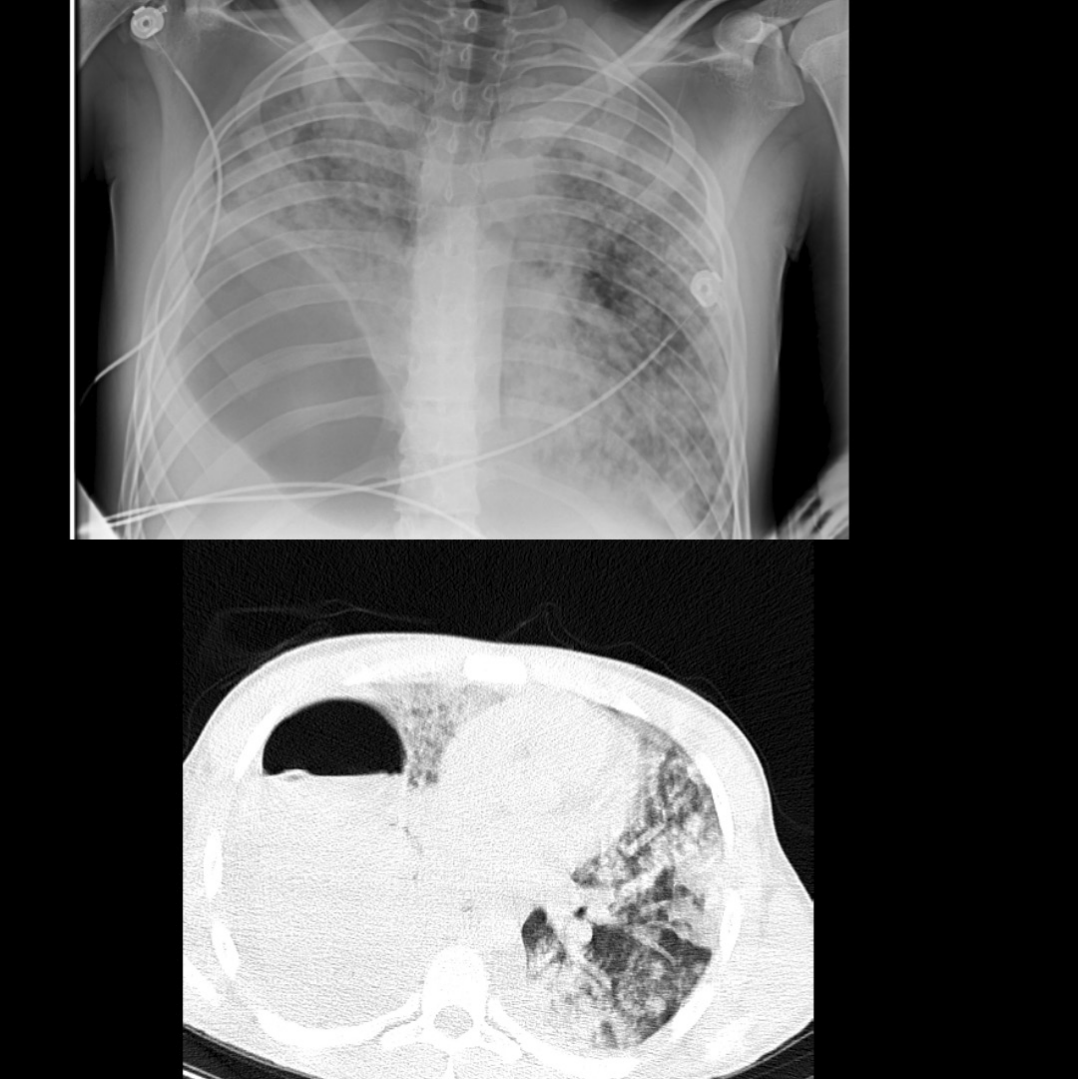
Chest X‐ray and Chest CT scan showing rupture of the cyst and diffuse ground glass opacities with centrilobular pattern in the field of both lungs suggesting ARDS.

He was hospitalized in the infectious disease ward for additional workup and treatment. Albendazole was administered 400 mg twice a day. A high titer of IgM antibody against echinococcus granulosus was detected in his serum. A thoracic surgery consult was done and surgery for resection of the cyst was planned. On the third day of admission, his oxygen saturation decreased to 70%, and he developed severe respiratory distress. Body temperature was 41 degrees Celsius and blood pressure was 100/65 mmHg. He was intubated and transferred to the intensive care unit (ICU). A Chest CT scan was performed and revealed rupture of the cyst and diffuse ground glass opacities with centrilobular pattern in the field of both lungs (Figure 2).

**FIGURE 3:**
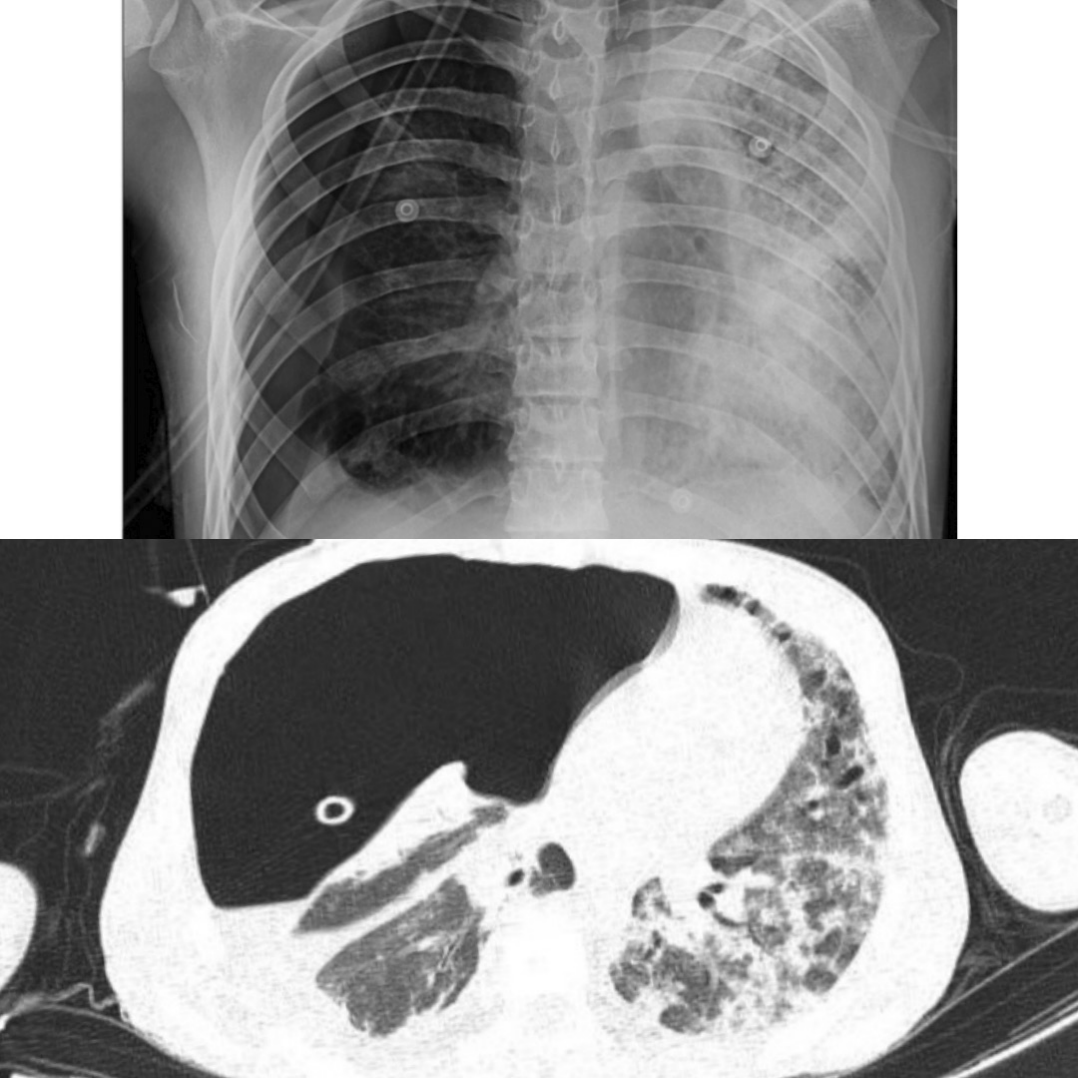
Chest X‐ray and Chest CT scan showing pneumothorax on the right lung with mediastinum shifted to the left.

Acute respiratory distress syndrome (ARDS) was suspected. Methylprednisolone 1 gr daily was started for 3 days followed by dexamethasone 4 mg three times a day intravenously. Broad‐spectrum antibiotic therapy was started for suspected secondary bacterial infection. Oral albendazole was continued. Five days later his oxygen saturation decreased and pneumothorax was detected on the chest CT scan. Therefore, a chest tube was placed in the right pleural cavity (Figure 3). Two weeks later he was extubated, and his oxygen saturation remained within normal limits by receiving oxygen through a facial mask. The chest tube was removed. He was discharged in stable condition four weeks after hospital admission. Oral albendazole was continued, and he was referred to a thoracic surgeon. He underwent resection of the cyst and lobectomy of the right middle lobe two weeks after discharge with no complications.

## 3 DISCUSSION

We reported a 17‐year‐old male presented with a large pulmonary hydatid cyst (PHC). Spontaneous rupture of the cyst led to acute respiratory distress syndrome requiring mechanical ventilation. Echinococcus granulosus, a parasitic tapeworm is the pathogenic organism of hydatid disease. This infectious disease is endemic in many parts of the world such as Iran and it is considered a health concern in these countries. While hydatid cysts could be produced in different organs, the liver and lungs are the most frequently affected sites. The definite hosts of the disease are carnivores such as dogs. Humans are defined as the incidental intermediate host who is infected by direct contact with dogs or ingestion of food contaminated by their feces. Echinococcal embryos are released in the gastrointestinal lumen and reach the blood vessels or lymphatics by penetrating the intestinal wall. Then they reach different organs including the liver and lungs through the bloodstream or lymphatics where they form hydatid cysts. As we mentioned in the case presentation section, our patient was a vegetable farmer and he might have been infected by the ingestion of contaminated vegetables.^3^, ^4^

Hydatid cyst remains asymptomatic during the first few years. Clinical presentation depends on the location and size of the cysts. The most frequently reported symptoms of pulmonary hydatid disease are cough, chest pain, dyspnea, and hemoptysis. Rupture of the cysts or secondary bacterial infection may occur. Rupture of the cysts either iatrogenic or spontaneous may cause cough, chest pain, hemoptysis, emesis, pneumothorax, pleural effusion, or empyema. Fever and hypersensitivity reactions including anaphylaxis may occur as a consequence of cyst rupture.^3^, ^4^

Chest X‐ray and chest CT scan are diagnostic tools for pulmonary hydatid cysts. Similar to our case, on chest CT scans, PHCs are smooth‐walled cysts with variable thickness and contain soft tissue with the density of water or near water. Leukocytosis, eosinophilia, and elevated ESR are nonspecific laboratory features. The coexistence of pulmonary and liver cysts is prevalent. Therefore, when PHC is diagnosed, additional imaging like ultrasonography should be done to investigate possible liver cysts. Surgery and medical treatment are two major therapeutic options. Surgery is the treatment of choice. Medical treatment includes benzimidazoles like mebendazole or albendazole which are administered orally and used for specific indications such as disseminated disease, contraindication for surgery, recurrent cysts, multiple cysts, or leakage of hydatid fluid during operation.^5^, ^6^

Fanne et al reported a 21‐year‐old female presented with productive cough, dyspnea, and fever. Two cavitary lesions with air‐fluid levels were found on the chest X‐ray. Five days after hospitalization she developed eosinophilia, ARDS, and anaphylactic shock and was intubated. Albendazole, praziquantel, and high doses of intravenous steroids were administered. She improved after four weeks and underwent cyst resection and lobectomy after discharge.^7^

In conclusion, acute respiratory distress syndrome could be a possible complication of a pulmonary hydatid cyst. Physicians should be aware of this life‐threatening complication for appropriate diagnosis and treatment.

## AUTHOR CONTRIBUTIONS

**Mehdi Salimi:** Conceptualization; writing – review and editing. **shirin assar:** Writing – original draft. **Dena Mohamadzadeh:** Writing – original draft. **Asal Kanjorpor:** Data curation.

## FUNDING INFORMATION

We received no funding.

## CONFLICT OF INTEREST STATEMENT

The authors declare that they have no competing interests.

## ETHICS STATEMENT

Approval was not needed by the local Clinical Research Ethics Committee for case reports.

## CONSENT

Written informed consent was obtained from the patient for publication of this case report in accordance with the journal's patient consent policy and any accompanying images. A copy of the written consent is available for review of the editor in chief of this journal.

## Data Availability

Data are available if requested.
